# Sampling Biases in Datasets of Historical Mean Air Temperature over Land

**DOI:** 10.1038/srep04637

**Published:** 2014-04-10

**Authors:** Kaicun Wang

**Affiliations:** 1 State Key Laboratory of Earth Surface Processes and Resource Ecology, College of Global Change and Earth System Science, Beijing Normal University, Beijing, 100875, China

## Abstract

Global mean surface air temperature (*T_a_*) has been reported to have risen by 0.74°C over the last 100 years. However, the definition of mean *T_a_* is still a subject of debate. The most defensible definition might be the integral of the continuous temperature measurements over a day (*T_d0_*). However, for technological and historical reasons, mean *T_a_* over land have been taken to be the average of the daily maximum and minimum temperature measurements (*T_d1_*). All existing principal global temperature analyses over land rely heavily on *T_d1_*. Here, I make a first quantitative assessment of the bias in the use of *T_d1_* to estimate trends of mean *T_a_* using hourly *T_a_* observations at 5600 globally distributed weather stations from the 1970s to 2013. I find that the use of *T_d1_* has a negligible impact on the global mean warming rate. However, the trend of *T_d1_* has a substantial bias at regional and local scales, with a root mean square error of over 25% at 5° × 5° grids. Therefore, caution should be taken when using mean *T_a_* datasets based on *T_d1_* to examine high resolution details of warming trends.

The measurements of the daily maximum and minimum temperatures (*T_max_* and *T_min_*) were developed in English-speaking countries once the maximum/minimum thermometers became widely used in approximately 1860^1^. The maximum (or minimum) thermometer is a unique thermometer in that its reading does not change after the air temperature (*T_a_*) reaches *T_max_* (or *T_min_*). Therefore, *T_max_* and *T_min_* can be easily measured by checking and resetting the thermometers once a day. The measurements of *T_max_* and *T_min_* have been accepted globally, and *T_d1_* = (*T_min_* + *T_max_*)/2 has already become the most common way to calculate mean *T_a_*^2^. In many weather stations, the measurements of *T_max_* and *T_min_* may be the only data source for historical mean *T_a_* and the only choice for a homogenous century-long analysis of mean *T_a_*^2^,^3^,^4^.

Analyses of global mean *T_a_* and its changes are performed operationally by several groups, including the NOAA National Climatic Data Center (NCDC) Global Historical Climatology Network (GHCN)^5^,^6^,^7^, the Goddard Institute for Space Studies (GISS)^8^, and a joint effort between the Met Office Hadley Center and the University of East Anglia Climate Research Unit (CRUTEM4)^9^,^10^. All of the global temperature analyses over land performed by the aforementioned groups rely heavily on *T_d1_*^11^,^12^. These century-duration analyses have provided basic datasets for global and regional climate change detection and attribution^13^.

However, the use of *T_min_* and *T_max_* to calculate mean *T_a_* has been criticised because *T_min_* is the response of a shallow nocturnal stable boundary layer^12^,^14^, and its variation is sensitive to local land use/land cover^15^, surface wind speed and humidity, and downward long-wave radiation from greenhouse gases^16^. *T_a_* reaches *T_min_* in the early morning because of long-wave cooling and reaches *T_max_* in the early afternoon because of solar short-wave radiation heating (Fig. 1). However, *T_a_* does not change linearly and symmetrically with time (Fig. 1); its diurnal curve depends on the proportion of surface absorbed energy partitioned into sensible and latent heat fluxes (or evapotranspiration)^17^, which is determined by the surface incident solar radiation, atmospheric downward long-wave radiation^16^, and surface aridity^18^. Therefore, *T_d1_* may deviate from real daily mean *T_a_* (i.e., *T_d0_*), for both climatology and long-term trends.

In this study, I make a first quantitative assessment of the bias of *T_d1_* in quantifying trends of mean *T_a_* using hourly *T_a_* observations collected by the NCDC Integrated Surface Database (ISD)^19^, which has provided long-term hourly *T_a_* measurements at approximately 5600 globally distributed weather stations since the 1970s (see Figs. S1 and S2 for detailed information).

## Results

The estimation of mean *T_a_* by *T_d1_* has two primary sources of bias: (1) *T_a_* has an asymmetric diurnal curve, and (2) *T_d1_* collects only two samples of *T_a_* from the early morning to the early afternoon and leaves roughly two thirds of a day without monitoring. The former introduces systematic bias to *T_d1_*, and the later primarily introduces random bias. These two sources of bias are evaluated separately in Figs. 2 and 3, respectively.

**Figure 1:**
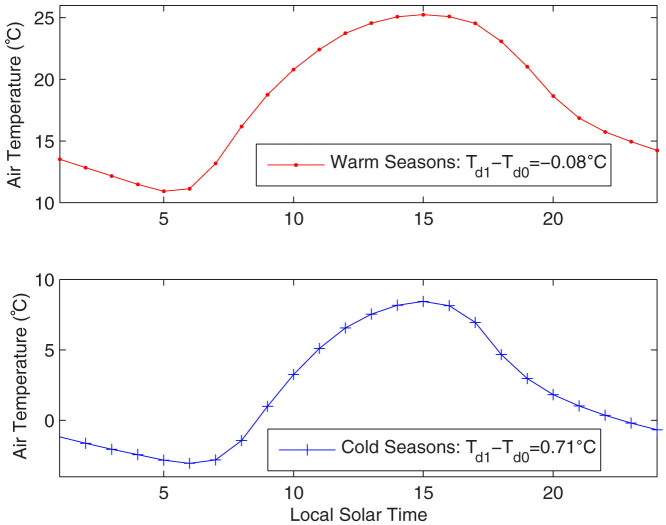
Multi-year averaged diurnal variation of air temperature (*T_a_*) during the warm seasons (May to October) and cold seasons (November to April) at a station (113.1°W, 37.7°N) in a semi-arid region in the U.S. Because of long-wave cooling, *T_a_* reaches its daily minimum, *T_min_*, in the early morning. The temperature reaches its daily maximum *T_max_* in the early afternoon because of solar radiation heating. *T_a_*, however, does not change linearly with time. During the cold seasons (Fig. 1b), *T_a_* reaches *T_max_* in the early afternoon but decreases quickly afterwards because surfaces receive less incident solar and long-wave radiation. Because of this asymmetric diurnal variation of *T_a_*, *T_d1_* = (*T_max_* + *T_min_*)/2 is 0.71 °C higher than *T_d0_*, integrated from the hourly temperature observations. However, *T_d1_* is almost equal to *T_d0_* during the warm seasons. The figure was produced using MATLAB.

**Figure 2:**
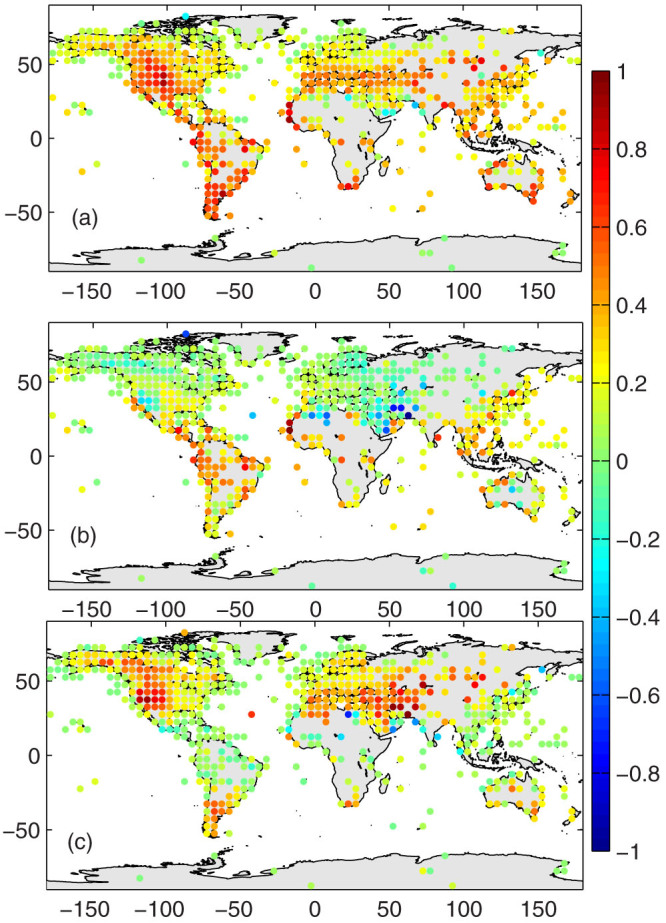
Multi-year averages of *T_d1_* − *T_d0_* in units of °C during (a) cold seasons (November to April in the Northern Hemisphere, or May to October in the Southern Hemisphere) and (b) warm seasons (May to October in the Northern Hemisphere, or November to April in the Southern Hemisphere). The differences between cold seasons and warm seasons are shown in panel (c). The 24-hour values of *T_a_* are calculated from the multi-year observations at each station. *T_d0_* is integrated from the 24-hour values, and *T_d1_* is averaged from the minimum and maximum of the 24-hour values. The climatology difference shown represents 5° × 5° grids, which are integrated from approximately 5600 weather stations. The figure was produced using MATLAB.

The 24-hour *T_a_* from multi-year observations at each station, as shown for example in Fig. 1, are comprised of hourly *T_a_* observations. *T_d0_* and *T_d1_* are calculated from these composite values. Their differences therefore reflect the impact of the shape of the diurnal variation of *T_a_* on the climatology of *T_d0_* and *T_d1_*. The mean surface air temperature *T_d1_* calculated from *T_max_* and *T_min_* has a substantially different climatology from that of the real mean surface air temperature *T_d0_* (Fig. 2), with a root mean square error of *T_d1_* − *T_d0_* of approximately 0.3 °C for global land measurements (Table 1). These differences in the climatologies between *T_d1_* and *T_d0_* are related to the asymmetric diurnal variation of *T_a_* (Fig. 1), which is determined by the surface energy balance. During cold seasons, *T_d1_* overestimates the mean surface air temperature *T_d0_* almost everywhere. This overestimation is higher in arid or semi-arid regions.

Two factors explain why *T_d1_* − *T_d0_* is much higher in cold seasons than in warm seasons in arid/semi-arid regions. First, in early morning, a larger fraction of the surface absorbed energy is partitioned into sensible heat flux during the cold seasons^17^ because the surfaces are covered by less vegetation and because it is drier in cold seasons than in warm seasons. These conditions result in a faster increase of *T_a_* in the morning under drier conditions because the sensible heat flux directly heats the air above the surface^18^. Second, in the afternoon, the surface long-wave cooling effect is more efficient (Fig. 1) in cold seasons because the compensating effect of atmospheric downward long-wave radiation is lower in the cold seasons due to their lower relative humidity^16^. The combination of these two factors results in higher values of *T_d1_* − *T_d0_* during the dry cold seasons in arid/semi-arid regions.

**Figure 3:**
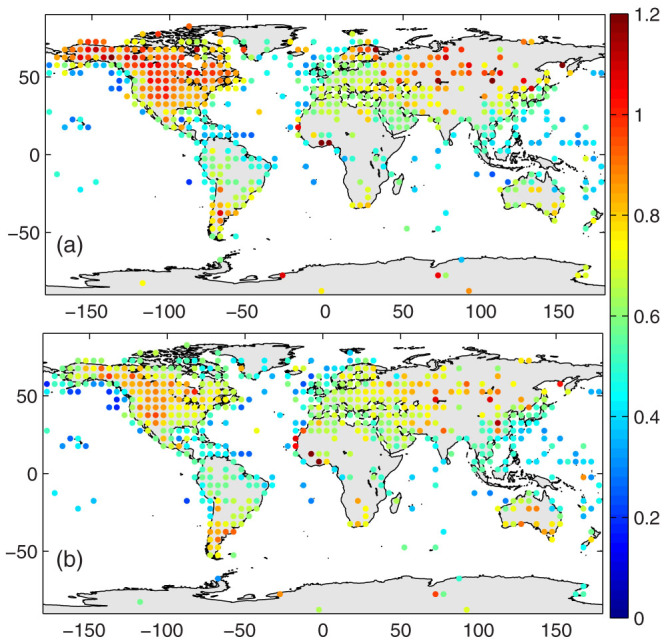
Standard deviation of the daily *T_d1_* − *T_d0_* (units: °C) in (a) cold seasons (November to April in the Northern Hemisphere, or May to October in the Southern Hemisphere), and (b) warm seasons (May to October in the Northern Hemisphere, or November to April in the Southern Hemisphere). *T_d1_* = (*T_max_* + *T_min_*)/2 is calculated from the daily maximum temperature (*T_max_*) and minimum temperature (*T_min_*), and *T_d0_* is integrated from hourly values during a day, defined as being from midnight to midnight. The standard deviations at stations are averaged to 5° × 5° grids. The standard deviations between daily *T_d1_* and *T_d0_* in winter seasons are generally larger than those in warm seasons. Spatially, the standard deviations between the daily *T_d1_* and *T_d0_* are higher in the northern high latitudes and the global arid/semi-arid regions (Eurasia, North America, South America, Africa, and Australia, see also Fig. S3). The figure was produced using MATLAB.

*T_d1_* samples *T_a_* only twice, during the early morning and the early afternoon, leaving approximately two-thirds of the day unmonitored. The unmonitored times may miss important information about the impact of weather events, such as fronts, on *T_a_* that are important during the cold seasons in the northern high latitudes. The deviation of *T_d1_* from *T_d0_* may be strong, but it is likely to be randomly distributed. The deviation may be quantified by calculating the standard deviation of the daily *T_d1_* − *T_d0_*. Fig. 3 confirms that the standard deviations of the daily *T_d1_* − *T_d0_* are higher at the northern high latitudes in the cold seasons and in the global arid/semi-arid regions in both cold and warm seasons. In general, the standard deviations of the daily *T_d1_* − *T_d0_* in the cold seasons are slightly larger than those in the warm seasons, with a mean value of approximately 0.6 °C (Table 1).

**Figure 4:**
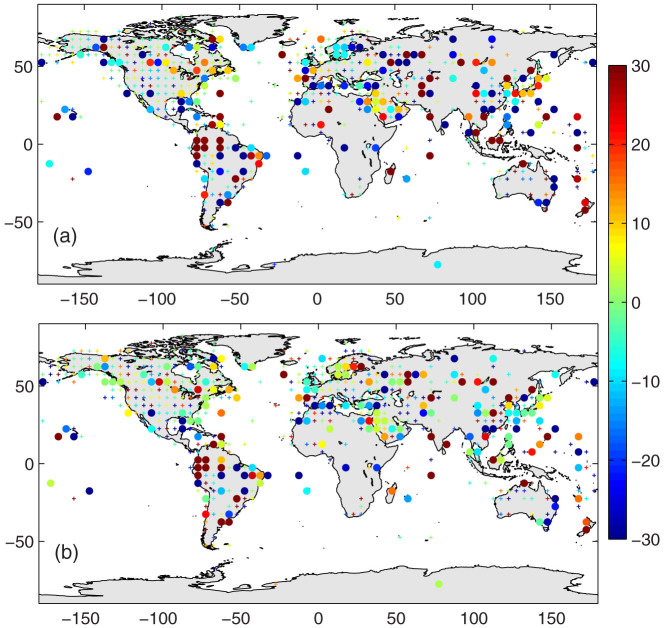
Bias (%) of the trend of *T_d1_* (or the trend of *T_d1_* − *T_d0_*) derived from the global weather observations collected by the NCDC ISD (Integrated Surface Database) and normalised by the trend of *T_d0_*: (a) cold seasons (November to April in the Northern Hemisphere, or May to October in the Southern Hemisphere), and (b) warm seasons (May to October in the Northern Hemisphere, or November to April in the Southern Hemisphere). The data shown here are integrated into 5° × 5° grids from approximately 5600 weather stations with different data periods for each station (see Fig. S2 for detailed information). *T_d1_* = (*T_max_* + *T_min_*)/2 is calculated from the daily maximum temperature (*T_max_*) and minimum temperature (*T_min_*), and *T_d0_* is integrated from the hourly values at the NCDC ISD stations. The dots indicate that the trends of *T_d1_* − *T_d0_* pass the α = 0.05 Student's t-test, and the small pluses indicate that the trends do not pass the confidence test. The positive values indicate that *T_d1_* overestimates the long-term trend of the mean surface air temperature. The figure was produced using MATLAB.

Figs. 2 and 3 show that the deviation of *T_d1_* from *T_d0_* depends on the asymmetric diurnal variation of *T_a_*, which is related to (a) the surface aridity and vegetation coverage and (b) the timing and frequency of weather events, i.e., front activity. Both aspects may change significantly under climate change conditions^20^,^21^,^22^, which may introduce a substantial bias to the trend of the mean surface air temperature through the use of *T_d1_*. Fig. 2 implies that the bias of *T_d1_* in quantifying the trend of the mean air temperature may be substantial in arid or semi-arid regions if the surface aridity or vegetation coverage changes. I, therefore, further assess the differences in the trends of *T_d1_* versus *T_d0_* using the NCDC ISD data. Fig. 4 shows the relative biases of the trends, and the statistical results are presented in Table 1. *T_d1_* has a negligible impact on the long-term trends of the mean surface air temperature, i.e., the global mean warming rate (Table 1).

The trends calculated from the monthly anomalies of *T_d1_*, however, have significant biases relative to *T_d0_* at the regional or local scales, with a root mean square error higher than 25% at a 5° × 5° grid scale (Table 1). The spatial coherence of biases in trends of the mean air temperature shown in Fig. 4 is minimal, in contrast with the results in Figs. 2 and 3. Three possible reasons may account for this minimal coherence: (1) the variable changes of the surface conditions occur in both direction and amount at different grids, (2) the measurement bias of the hourly *T_a_* that were used to calculate *T_max_*, *T_min_*, *T_d1_* and *T_d0_*, and (3) the different time periods of data at different grids in Fig. 4, as shown in Fig. S2.

## Discussion

In this study, the maximum and minimum hourly *T_a_* measurements are selected to represent *T_max_* and *T_min_*. However, the response time of the *T_max_* and *T_min_* thermometer is several minutes. This may introduce some bias to the conclusions of this study. Here, I use the *T_a_* data of five-minute resolution collected by the U.S. Climate Reference Network (USCRN) to demonstrate that it is reliable to use hourly *T_a_* measurements to represent *T_max_* and *T_min_*.

USCRN has operated since the year 2000 at a few sites and was officially and nationally commissioned by the National Oceanic and Atmospheric Administration (NOAA) in 2004^23^,^24^. The primary goal of USCRN is to provide long-term high-quality homogeneous observations, in particular, for *T_a_* and precipitation^25^; its temperature system consists of a platinum resistance thermometer and an aspirated radiation shield^24^,^25^. At each site, the USCRN uses three such thermometers for inter-comparison and quality assurance^25^. These redundant high-level thermometers at each USCRN site guarantee their high-quality continuous observations of *T_a_* (the missing rate of its *T_a_* data is near zero). The data sampling rate of the USCRN thermometers is 5 seconds, and temperature signals averaged over 5-minute intervals are output.

**Figure 5:**
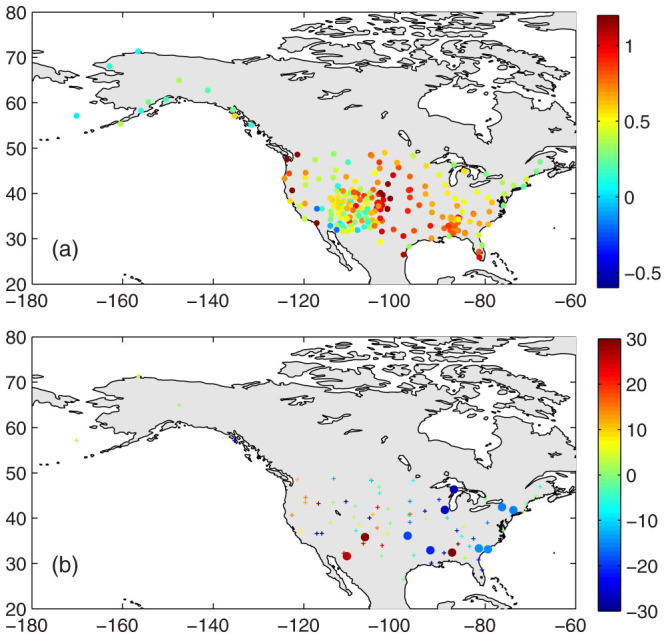
Multi-year averages of (a) *T_d1_* − *T_d0_* in units of °C, and (b) the trend of *T_d1_* − *T_d0_* normalised by the trend of *T_d0_* in units of %. The observations of the air temperature at five-minute temporal resolution collected by USCRN from 2004 to 2013 are used here. *T_d0_* is integrated from the five-minute values from midnight to midnight, and *T_d1_* is averaged from the minimum and maximum of the five-minute values. The dots indicate that the trends of *T_d1_* − *T_d0_* pass the α = 0.05 Student's t-test, and the small pluses indicate that the trends do not pass the confidence test. Each symbol represents a USCRN site. The positive values indicate that *T_d1_* overestimates the long-term trend of the mean surface air temperature. The biases are limited to ±30%, avoiding normalisation by near-zero trends. The figure was produced using MATLAB.

USCRN provides a method for coupling its continuous measurements to the past observations^24^,^26^. *T_max_* and *T_min_* can be directly determined from its 5-minute averages^24^,^26^, from which *T_d1_* is calculated. We processed all of the data in local solar time. Monthly averages are calculated only if the daily means are available on no less than 24 days in a month and if there are no gaps of four or more consecutive days. The trends of the *T_d0_* and *T_d1_* are calculated from the monthly anomalies after removing their seasonal cycles. Fig. 5 shows that the biases in the mean values of *T_d1_* using the USCRN data are similar to those from the ISD, both in amount and spatial variability. The biases in the relative trend of *T_d1_* using the USCRN data are of similar magnitude to those using the ISD data, but with different spatial pattern because the time durations of the two datasets are different. As data availability of the USCRN is temporally and spatially less than that of ISD, the main results in this study are reported using the ISD data.

What are the implications of the biases of *T_d1_* and of its trend uncovered in this analysis? It was reported that the warming rate was stronger in the cold-season in semi-arid regions using observations based on *T_d1_*^27^. Observations of global mean temperature that are primarily based on *T_d1_*, including CRUTEM3, GISS, and NCDC, were used to evaluate *T_d0_* from the simulation of the Intergovernmental Panel on Climate Change Fourth Assessment Report (IPCC AR4) climate models. It was found that the model simulations of *T_d0_* did not simulate the full extent of the observed winter time warming of *T_d1_* at the high-latitude Northern Hemisphere^28^. As shown in Fig. 2, *T_d1_* will overestimate the trend of mean *T_a_* if the area gets drier in the arid/semi-arid regions. Observed evidences show it is the case as middle latitude arid or semi-arid regions have been drier in recent decades^29^,^30^. This partly explains the enhanced warming in semi-arid regions in cold seasons.

Although mean temperature, *T_d1_* = (*T_max_* + *T_min_*)/2, is perhaps the most common method used to calculate mean air temperature, it is the not the only option. Scandinavian countries developed a special formula to estimate mean *T_a_*^31^. For example, Sweden still uses the Ekholm–Modén formula from 1916, in which the mean *T_a_* is a linear combination of the *T_max_*, *T_min_*, and measurements of *T_a_* at 6, 12, and 18 h UTC^31^. Another option is to calculate the mean surface air temperature from *T_a_* measurements at 00, 06, 12 and 18 h UTC (*T_d2_*), which are recently available through the World Meteorological Organization's (WMO) global telecommunication system^32^. These data have been used in climate-related research^33^.

The significant differences in the climatology of *T_d1_* and *T_d0_* (or *T_d2_*), as shown in Fig. 2 (or Fig. S3), preclude switching from the use of *T_d1_* to *T_d0_* (or *T_d2_*) for estimating the long term variability of the mean surface air temperature, although *T_d0_* or *T_d2_* is already globally available (Figs. 2 and S2). To produce a century-long homogenous dataset of mean *T_a_*, it is essential to continue to use the measurement methods currently used at the weather stations^3^,^4^. This study indicates that the use of *T_d1_* has a negligible impact on the global mean warming rate. However, *T_d1_* cannot accurately reflect the impact of the changes in the surface conditions on the variability of *T_a_*. These changing surface conditions cause the diurnal curve of *T_a_* to vary with time, thereby resulting in the deviation of *T_d1_* from *T_d0_*. Therefore, the trend of *T_d1_* has a substantial bias at regional and local scales, with a root mean square error of over 25% for 5° × 5° grids. Careful attention should be paid when using mean surface air temperature *T_d1_* on studies regarding the spatial patterns of warming. For such studies, recently available hourly measurements^19^ are recommended.

Factors that can introduce inhomogeneity to the mean *T_a_* have been reviewed by Jones et al.^34^. In particular, by assuming that a departure from differently derived mean values are comparable, Bradley and Jones^35^ inferred that the monthly temperature values and the hemispherical estimates from different definitions were the same if calculated as anomalies from a selected period. This study confirms this assumption by demonstrating that the global mean trend calculated from the monthly anomalies of *T_d1_* is the same as that of *T_d0_*. However, the trends calculated from the monthly anomalies of *T_d1_* at 5° × 5° grids have a significant error, with a standard deviation of 25%. This large error is caused by the variation of *T_d1_* − *T_d0_* with changing surface conditions (Fig. 2) as a result of the changing diurnal curve of *T_a_* (Fig. 1).

The use of *T_max_* and *T_min_* to calculate the mean *T_a_* over land is a result of the fact that many places have used inexpensive instruments that only measure those two temperatures (and could only be checked by an observer once a day) for a long time. *T_min_* is more sensitive to changes in land cover^15^ and atmospheric downward long-wave radiation^12^, while the diurnal temperature range (*T_max_* − *T_min_*) is more sensitive to changes in land-atmosphere turbulent fluxes^17^, which are driven by variations in the surface incident solar radiation^18^ caused by the changes in the clouds and aerosols^36^,^37^. Observations from *T_max_* and *T_min_* are therefore very important because of their relationship to climate change impacts and their connection to the global energy balance.

## Methods

The hourly observations of *T_a_* over global land collected by the NCDC ISD project^19^ were used in this study. The ISD compiles data from over 100 original data sources that archive hundreds of meteorological variables. The ISD has archived 2 billion surface weather observations from over 20,000 stations worldwide from 1900 to the present. Currently, the ISD database is updated with observations from over 11,000 active stations on a daily basis.

The ISD provides consistent and standardised quality control of the global hourly meteorological observations^19^. The ISD contains 54 quality control (QC) algorithms that serve to process each data observation through a series of validity checks, extreme value checks, internal consistency checks, and external continuity checks. Among all of the parameters, temperatures are among the most extensively validated parameters. The ISD data can be freely downloaded from www.ncdc.noaa.gov/oa/climate/isd/index.php.

As of August 2013, there were approximately 5600 stations reporting hourly *T_a_* measurements for more than five years (Fig. S1). The ISD data were reported in UTC time and converted into local solar time for the purpose of my analysis. To obtain the bias and to reduce the impact of missing data, I produced a composite of the 24 hourly values at each site, i.e., all of the observations were averaged into hourly values, and the 24-hour values of the *T_a_* were obtained for each site. *T_min_* and *T_max_* were selected from the 24-hour values, from which *T_d1_* was calculated. *T_d0_* was integrated from the 24-hour values. In this study, I split a year into cold seasons (November to April in the Northern Hemisphere, or May to October in the Southern Hemisphere) and warm seasons (May to October in the Northern Hemisphere, or November to April in the Southern Hemisphere). The climatological differences of *T_d1_* and *T_d0_* were aggregated into a 5° × 5° grid and are shown in Fig. 2. The composite method used here substantially reduces the impact of missing data on the results in Figs. 1 and 2. If there are no missing data, the results shown in Figs. 1 and 2 should be equal to those based on the daily basis, provided that the day is defined as being from midnight to midnight.

The trends shown in Fig. 4 were calculated differently. *T_min_* and *T_max_* were first selected from the 24-hour observations for each day at every site. The data were regarded as reliable only if the hourly temperatures were available for more than 22 h a day, from which the daily and monthly *T_d0_* and *T_d1_* were calculated. The monthly values were regarded as reliable only if the daily values were available for more than 15 days a month. The requirement for hourly air temperature measurements is stricter than that for daily values because this study focuses on the difference of *T_d0_* and *T_d1_*, which is dominated by the diurnal curve of air temperature. The monthly anomalies of *T_d1_* and *T_d0_* were calculated at each weather station by removing their averaged seasonal cycle. The monthly anomalies were then aggregated into 5° × 5° grid values. The grid averaged-monthly anomalies were regarded as reliable if the data for each month was available at more than 50% of the stations within the grid. The trends of *T_d0_* and *T_d1_* − *T_d0_* calculated from the grid monthly anomalies are presented in Fig. 4 and Table 1. The data duration of *T_d1_* and *T_d0_* at the 5° × 5° grid can be found in Fig. S2.

The measurements of *T_min_* and *T_max_* were developed in English-speaking countries where the maximum/minimum thermometers were widely used since approximately 1860^1^. A maximum thermometer is a unique mercury thermometer that functions by having a constriction in the neck close to the bulb. The mercury is forced up through the constriction by the force of expansion as the temperature increases. When there is a decrease in the temperature, the volume of mercury contracts but cannot return to the bulb because of the narrow of the bulb neck. As a result, the column of mercury breaks at the constriction and remains stationary in the tube. The minimum thermometer works similarly, but it does so with steel pin immersed in clear liquid (i.e., ethyl alcohol) in glass.

The measurements of *T_max_* and *T_min_* can be made by one visit to the weather station a day. Because of its low cost, the measurements of *T_max_* and *T_min_* have been accepted globally, and *T_d1_* = (*T_min_* + *T_max_*)/2 has become the most common method to calculate the mean surface temperature. For most weather stations, the measurements using this method may be the only data source for historical temperature.

The weather stations of ISD directly measured *T_max_* and *T_min_*, in addition to the hourly temperature. The *T_max_* and *T_min_* temperatures were defined as the highest and lowest temperatures to have occurred during the past 24 hours. However, the *T_max_* or *T_min_* measurements may depend on the observation schedule, which may be different from country to country. The definition of a day is therefore different, such as from midnight to midnight or from noon to noon. In Europe, *T_min_* and *T_max_* are usually reported for 12-hour intervals ending at 6 UTC and 18 UTC and are not necessarily the true *T_min_* and *T_max_* in many regions, especially during the winter months^38^. This discrepancy occurs because 6 UTC is before the climatologically coldest hour sunrise in the winter in some regions (i.e., Europe)^33^ and is also partly a result of the synoptic weather variability. This discrepancy introduces a significant error in the estimations of daily *T_max_* and *T_min_*^33^. The changes of the observation schedules may also introduce inhomogeneity of the climatology of the surface mean air temperature^38^. These problems can be avoided either by maintaining an unchanged observation schedule at a station or by using hourly observations, as I did here.

The bias caused by changes to the observation schedules of *T_min_* and *T_max_* may be important^15^,^38^, but are not discussed here because the information on observation schedules is not yet publicly available. Two primary sources of bias of *T_d1_* are discussed in this study (Figs. 2 and 3). They have physical meanings and may introduce substantial bias to trends of mean surface air temperature *T_d1_*. The differences among the trends of *T_d1_* and *T_d0_* (Fig. 4), which are much less sensitive to the definition of the day, are calculated using the definition of day as midnight to midnight.

## Supplementary Material

Supplementary Information SUPPLEMENTARY INFORMATION

## Figures and Tables

**Table 1:** Statistical parameters of the sampling bias of mean surface air temperature *T_d1_* = (*T_max_* + *T_min_*)/2. The mean temperature *T_d0_* is integrated from the hourly values. *T_max_* and *T_min_* are selected from the hourly values. The data used in the table are hourly observations collected by the NCDC Integrated Surface Database (ISD) over global land. The climatology difference and standard deviation of daily *T_d1_* − *T_d0_* have units of °C, and the trend of *T_d1_* − *T_d0_* is in units of °C per decade. The numbers in brackets indicate their relative values

		Average	Median	Root Mean Square
Cold Seasons	Climatology difference of *T_d1_* − *T_d0_*	0.30	0.28	0.36
	Standard deviation of Daily *T_d1_* − *T_d0_*	0.67	0.67	0.70
	Trend of *T_d1_* − *T_d0_*	−0.01 (2.7%)	−0.01 (−1.4%)	0.06 (25.5%)
Warm Seasons	Climatology difference of *T_d1_* − *T_d0_*	0.11	0.09	0.24
	Standard deviation of Daily *T_d1_* − *T_d0_*	0.61	0.63	0.64
	Trend of *T_d1_* − *T_d0_*	0.00 (−1.5%)	−0.00 (−1.0%)	0.06 (27.1%)
