# Supplementary material for: Sampling Biases in Datasets of Historical Mean Air Temperature over Land
Source: Sci Rep. 2014 Apr 10;4:4637. doi: 10.1038/srep04637 (PMC3982162; doi:10.1038/srep04637)
Supplement: Supplementary Information [file srep04637-s1.pdf]

SUPPLEMENTARY INFORMATION

Sampling Biases in Datasets of Historical Mean Air Temperature over Land

Kaicun Wang

State Key Laboratory of Earth Surface Processes and Resource Ecology, College of Global Change and Earth System Science, Beijing Normal University, Beijing, 100875, China

Corresponding Author: Kaicun Wang, State Key Laboratory of Earth Surface Processes and Resource Ecology, College of Global Change and Earth System Science, Beijing Normal University. Email: kcwang@bnu.edu.cn; Tel: 86+ 10-58803143; Fax: 86+10-58800059.

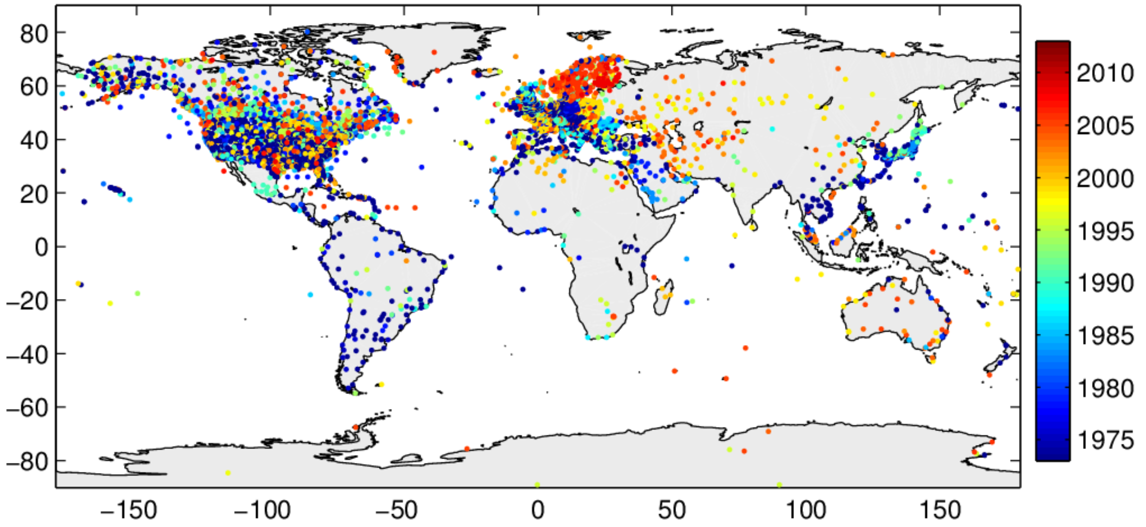

Fig. S1. A map that presents the locations where the hourly measurements of the air temperatures were performed and for which the data are available for more than five-years in the NCDC ISD database. The colour shown in this figure indicates the start year of the hourly measurements at the stations. The figure was produced using MATLAB.

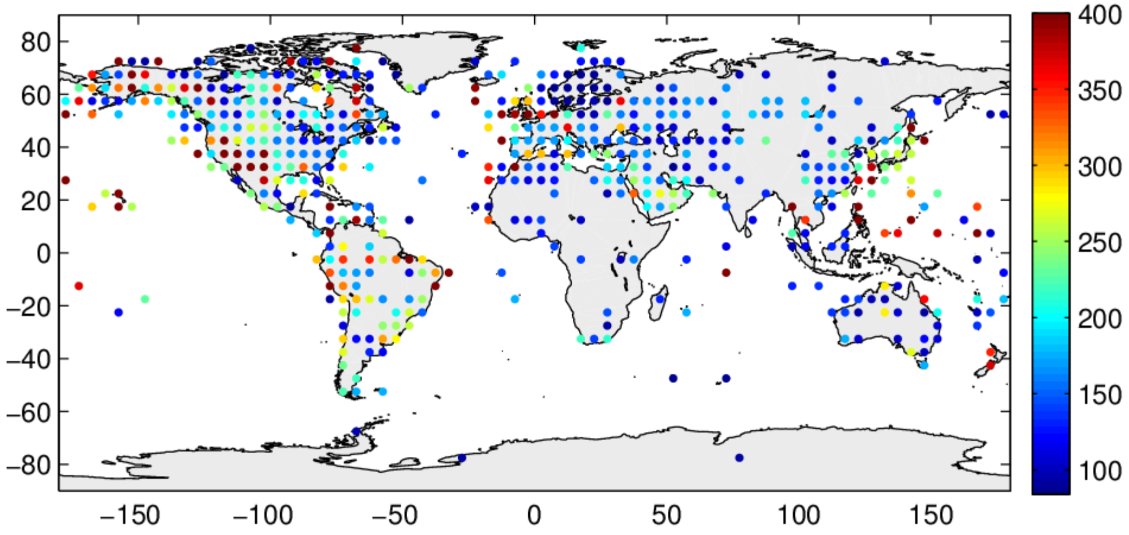

Fig. S2. A map showing the data duration of the hourly air temperature at the 5° × 5° grid (units in the map: month). The monthly anomalies of the NCDC ISD are aggregated into the 5° × 5° grids. The average of the monthly anomalies are regarded as reliable if the data of the month are available at more than 50% of all of the stations within the grid. The colour shown in this figure indicates the data duration. Fig. 4 and Table 1 are based on the data shown here. Some stations in the U.S. and Europe have hourly measured air temperature data since before the 1970s. The figure was produced using MATLAB.

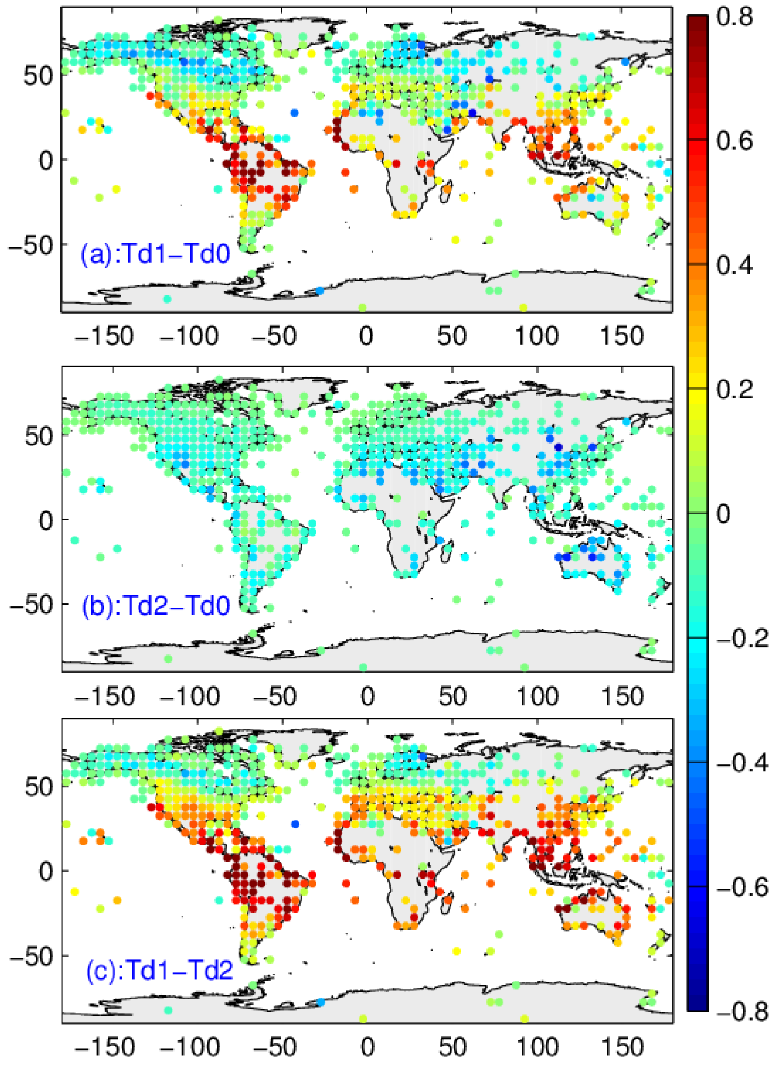

Fig. S3. Multi-year averages of (a) T_{d1}-T_{d0}, (b) T_{d2}-T_{d0}, and (c) T_{d1}-T_{d2} in units of °C. T_{d0} is integrated from the 24-hour values from midnight to midnight, T_{d1} is averaged from the minimum and maximum of the 24-hour values, and T_{d2} is averaged from T_a at 00:00, 06:00, 12:00, and 18:00, as recommended by the World Meteorological Organization (WMO). The climatology differences are shown in 5° × 5° grids, which are integrated from approximately 5600 weather stations. The absolute values shown in figure depend on the definition of the day, which is here defined as midnight to midnight of the local solar time. The figure was produced using MATLAB.
